# Association of CHA2DS2-VASc Score with Long-Term Incidence of New-Onset Atrial Fibrillation and Ischemic Stroke after Myocardial Infarction

**DOI:** 10.3390/jcm11237090

**Published:** 2022-11-30

**Authors:** Samuli Jaakkola, Tuomas Paana, Juhani Airaksinen, Jussi Sipilä, Ville Kytö

**Affiliations:** 1 Heart Center, Turku University Hospital, University of Turku, 20521 Turku, Finland; 2 Department of Neurology, Siun Sote, North Karelia Central Hospital, 80210 Joensuu, Finland; 3 Turku Clinical Research Center, Turku University Hospital, 20521 Turku, Finland; 4 Center for Population Health Research, Turku University Hospital, University of Turku, 20521 Turku, Finland

**Keywords:** myocardial infarction, atrial fibrillation, ischemic stroke, cha2ds2-vasc

## Abstract

The CHA_2_DS_2_-VASc score is a reliable tool used to estimate the risk of ischemic stroke (IS) in patients with atrial fibrillation (AF). Few tools exist for the prediction of new-onset AF (NOAF) after myocardial infarction (MI) and its relation to IS. We studied the usefulness of CHA_2_DS_2_-VASc in predicting NOAF and IS in a long-term follow-up after MI. Consecutive MI patients without baseline AF (*n* = 70,922; mean age: 68.2 years), discharged from 20 hospitals in Finland during 2005–2018, were retrospectively studied using national registries. The outcomes of interest after discharge were NOAF- and IS-assessed with competing risk analyses at one and ten years. The median follow-up was 4.2 years. The median baseline CHA_2_DS_2_-VASc score was 3 (IQR 2–5). The likelihood of both NOAF and NOAF-related IS increased stepwise with this score at one and ten years (all *p* < 0.0001). The one-year-adjusted subdistribution hazard ratio (sHR) was 4.03 (CI 3.68–4.42) for NOAF in patients with CHA_2_DS_2_-VASc scores ≥6 points. The cumulative incidence of IS was 15.2% in patients with NOAF vs. 6.2% in patients without AF at 10 years after MI (adj. sHR 2.12; CI 1.98–2.28; *p* < 0.0001). Coronary artery bypass surgery was associated with a higher NOAF incidence compared to percutaneous coronary intervention (adj. sHR 1.87; CI 1.65–2.13; *p* < 0.0001 one year after MI). The CHA_2_DS_2_-VASc score is a simple tool used to estimate the long-term risk of NOAF and IS after MI in patients without baseline AF. Coronary bypass surgery is associated with an increased NOAF incidence after MI.

## 1. Introduction

Patients suffering from myocardial infarction (MI) are known to be at an increased risk of atrial fibrillation (AF) [1,2]. New-onset AF (NOAF) developing after MI is associated with an excess risk of death, ischemic stroke (IS), and impaired quality of life [3,4]. While the risk factors for AF and IS are well established in the general population, there is little information on the predictors and clinical outcomes of NOAF after MI [5]. Further, the influence of different types of revascularization on the occurrence of NOAF and IS has not been thoroughly studied. The CHA2DS2-VASc score is a simple, easily applicable tool used to predict IS risk in AF patients [6]. Considering the elevated risk of IS associated with AF, it is important to identify patients at risk of developing NOAF after MI. We studied the usefulness of the CHA2DS2-VASc score in predicting NOAF and IS in a long-term follow-up after MI. In addition, we studied the association of revascularization with NOAF and IS.

## 2. Materials and Methods

### 2.1. Patients and Design

Data on all consecutive MI patients admitted to participating hospitals in Finland between 1 January 2005 and 30 June 2018 who were discharged alive were retrospectively collected from the Care Register for Health Care in Finland (CRHC). All Finnish hospitals that treat MI patients (*n* = 20, including five university hospitals with coronary surgery available) were included [7]. The index MI was identified based on ICD-10 code I21 as the primary discharge diagnosis, and only initial MI admissions during the study period to medical (including cardiology), surgical (including cardiac surgery), and intensive care wards were included [8]. In-hospital and between-hospital transfers were combined as a single admission. The present study focused only on patients with AF first diagnosed after MI. Therefore, patients with an AF diagnosis (ICD-10 code I48) before or during an index MI admission were excluded. In addition, patients treated with an oral anticoagulant prior to MI, patients treated with valvular or aortic surgery during index admission, and patients without follow-up data (0.6%) were excluded (Supplementary Figure S1). Prior oral anticoagulant treatment was defined as a prescription purchase within 90 days prior to MI [8].

Baseline congestive heart failure, hypertension, diabetes, and stroke/transient ischemic attack/ischemic thromboembolism were detected [9] (Supplementary Materials and Supplementary Table S1). Age at the index MI was classified as <65, 65–74, and ≥75 years. The CHA_2_DS_2_-VASc score at baseline was calculated and classified as 1, 2, 3, 4, 5, and ≥6 points. One CHA_2_DS_2_-VASc score point was calculated for all patients (due to MI), as well as one additional point for those with congestive heart failure, hypertension, being aged 65–75, diabetes, and the female sex. Two additional points were calculated for those aged ≥75 and with stroke/TIA/ischemic thromboembolism. Revascularization procedures during index MI hospitalization and the type of MI (ST level changes) were recorded [10].

### 2.2. Outcomes and Follow-Up

The outcomes of interest were NOAF and NOAF-related IS assessed at a one- and ten-year follow-up after index event (MI for NOAF and NOAF for NOAF-related IS). The outcomes are detailed in the Supplementary Materials. The follow-up ended on 31 December 2018. Outcomes were determined based on a combination of the CRHF registry and the cause of death registry [11].

### 2.3. Data Sources

The CRHC registry data, including data on all hospital admissions and major interventional procedures, and special reimbursement entitlement data were obtained from Findata (permission no: THL/164/14.02.00/2021). Pharmacy purchases of oral anticoagulants before MI were obtained from a national drug purchase database (THL/164/14.02.00/2021). Mortality and cause-of-death data were obtained from Statistics Finland (TK-53-484-20). The included registries are mandated by law and cover the entire Finnish population. Ethical board review and informed consent were waived due to the study design, and the participants were not contacted. The legal basis for the processing of personal data is public interest and scientific research (EU General Data Protection Regulation 2016/679 (GDPR), Article 6(1)I and Article 9(2)(j); Data Protection Act, Sections 4 and 6).

### 2.4. Statistical Analysis

The outcomes were studied using the cumulative incidence function and Fine–Gray regression, accounting for the competing risk of non-endpoint-specific death [12]. Schoenfeld residuals were used for confirmation of proportional subdistribution hazard (sHR) assumptions. Dichotomic trends were studied using the Cochrane–Armitage trend test. The results were given as the mean, median, percentage, or subdistribution hazard ratio with a 95% confidence interval (CI), interquartile range (IQR), or ±SD. Statistical significance was inferred at a *p*-value < 0.05. Analyses were performed with SAS version 9.4 (SAS Institute, Inc., Cary, NC, USA).

## 3. Results

The final study cohort included 70,922 hospital-surviving MI patients. The mean age was 68.2 years, and 34.7% were female. The median CHA_2_DS_2_-VASc score was 3 (range 1–9; IQR 2–5). Percutaneous coronary intervention (PCI) was performed on 55.3% of the patients and a coronary artery bypass (CABG) was performed on 7.5%, while 37.3% were treated conservatively (Table 1). The median follow-up was 4.2 (IQR 1.8–7.7) years for NOAF incidence and 4.6 (IQR 2.1–8.1) years for NOAF-related IS.

### 3.1. New-Onset Atrial Fibrillation

**Figure 1:**
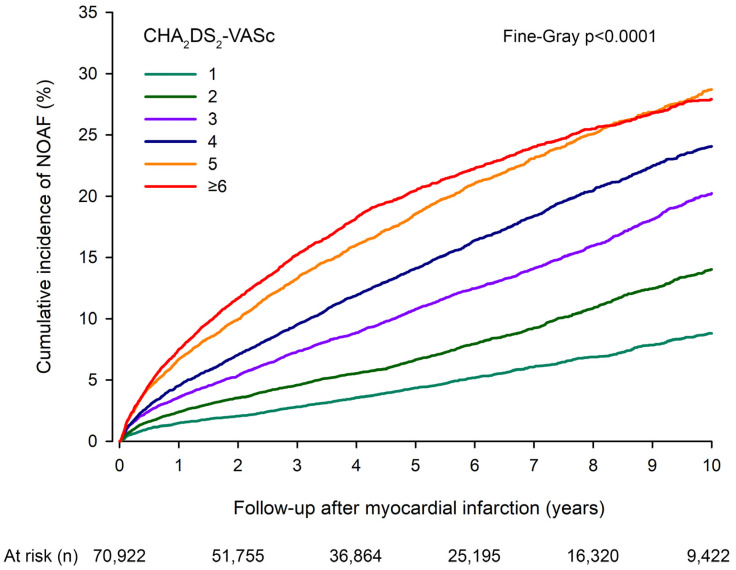
Cumulative incidence of new-onset atrial fibrillation (NOAF) after myocardial infarction by CHA2DS2-VASc score.

NOAF was diagnosed in 9614 patients during the follow-up after MI. The cumulative incidence of NOAF in the total study cohort was 4.1% at the one-year follow-up and 19.8% at the ten-year follow-up after MI. The median time from MI to NOAF was 2.5 years (IQR 0.8–5.2 years). An increased CHA_2_DS_2_-VASc score was associated with an increased incidence of NOAF both at one and ten years (*p* < 0.0001 for both; Table 2). After MI (Figure 1), the one-year-adjusted sHR was 4.66 (CI 3.92–5.55) and the ten-year-adjusted sHR was 4.03 (3.68–4.42) for NOAF in patients with the highest score (≥6 points) compared to patients with the lowest score (1 point) (Supplementary Table S2).

Revascularization by PCI was associated with a decreased NOAF incidence compared to non-revascularized patients at one year (3.0% vs. 5.4%; adj. sHR 0.75; CI 0.68–0.82; *p* < 0.0001) and ten years (17.6% vs. 21.6%; adj. sHR 0.76; CI 0.71–0.82; *p* = 0.014). Conversely, CABG was associated with an increased NOAF incidence compared to non-revascularized patients at both the one-year (5.9% vs. 5.4%; adj. sHR 1.40; CI 1.23–1.59; *p* < 0.0001) and ten-year (24.2% vs. 21.6%; adj. sHR 1.24; CI 1.15–1.33; *p* < 0.0001) time points. Compared to patients revascularized with PCI, CABG was associated with a higher incidence of AF at one year (5.9% vs. 3.0%; adj. sHR 1.87; CI 1.65–2.13; *p* < 0.0001) and at ten years (24,2% vs. 17.6%; adj. sHR 1.31; CI 1.22–1.41; *p* < 0.0001) after MI. The incidence of NOAF within one year after MI did not change significantly during the study period (Supplementary Table S2).

### 3.2. Ischemic Stroke

IS occurred in 3791 patients during the 10-year follow-up (cumulative incidence 7.8%). Of all IS cases, 853 (22.5%) were associated with NOAF. In addition, NOAF was associated with an increased IS occurrence both in univariable (sHR 2.59; CI 2.42–2.77; *p* < 0.0001) and multivariable (adj. sHR 2.12; CI 1.98–2.28; *p* < 0.0001) analyses of the follow-ups of all study patients. NOAF was diagnosed simultaneously with IS in 4.1% of all NOAF patients and in 46.1% of NOAF-associated IS cases, with a median time of six days (IQR 0 days–1.7 years) between NOAF diagnosis and IS. The cumulative incidence of IS was 6.4% at one year and 10.9% at ten years after NOAF diagnosis. A higher CHA_2_DS_2_-VASc score was associated with a higher proportion of NOAF-associated IS (trend *p* = 0.001) (Table 3). The incidence of IS increased stepwise with the CHA_2_DS_2_-VASc score both at one year and ten years after NOAF diagnosis (*p* < 0.0001 for both; Table 4). The adjusted sHR for IS after NOAF in the highest CHA_2_DS_2_-VASc score group was 1.95 (1.27–2.99) at one year and 1.85 (1.31–2.62) at ten years compared to the lowest score group (Supplementary Table S3). Revascularization by PCI was associated with a lower incidence of IS in patients with NOAF during the follow-up when compared to no revascularization (adj. sHR 0.81; CI 0.70–0.94; *p* = 0.007), but there was no difference in the CABG and no-revascularization groups (adj. *p* = 0.990; Supplementary Table S3).

## 4. Discussion

Our results found that the CHA_2_DS_2_-VASc score is a feasible tool used to predict NOAF during a long-term follow-up in patients with a history of MI. The hazard of developing NOAF within one year after MI was 4.6-fold higher in those with the highest CHA_2_DS_2_-VASc score (6 points) than in those with the lowest score (1 point). Importantly, the occurrence of NOAF after MI was associated with an increased risk of IS. The IS subdistribution hazard was 2.1-fold higher in MI survivors who developed NOAF during the study follow-up. Despite the potential influence of cofounding factors, our results suggest PCI to be the more favorable method for revascularization in relation to NOAF occurrence, as CABG was associated with a higher NOAF incidence at one year (1.9-fold) and, interestingly, at ten years (1.3-fold) as well.

A higher NOAF rate after MI compared to the general population has been well documented, with incidences ranging from 6% to 21% at five years post-MI [13]. Our findings are similar, as the NOAF rates were 4.1% within one year and 19.8% within ten years after MI. MI can cause irreversible structural and hemodynamic changes that manifest as clinical arrhythmias, most commonly within the first year after MI [3]. This temporal trend was also observed in our study. However, myocardial scarring may also act as a substrate for arrhythmias after index MI, resulting in the high incidence of NOAF observed in our long-term follow-up [14].

Several independent risk factors for AF have been identified in the general population, but fewer have been identified for MI survivors [5]. Most studies address NOAF occurrence during MI hospitalization or compare patients with a history of AF and those who develop AF during follow-up [4]. A recent study found that a history of angina, worse patient-reported quality of life, European origin, and a bleeding event prior to index MI all predicted AF incidence over two years of follow-up [4]. The focus of our study was the investigation of the applicability of the commonly used CHA_2_DS_2_-VASc score used to predict NOAF in patients with a history of either STEMI (ST-elevation myocardial infarction) or non-STEMI. Our results show that the CHA_2_DS_2_-VASc score can indeed be used to identify at-risk patients to yield more targeted follow-ups and rhythm monitoring to detect AF. In 2014, Lau et al. studied the usefulness of the CHA_2_DS_2_-VASc score in predicting NOAF and IS in post-STEMI patients [15]. Conversely, our study results apply to all MI survivors, covering a much larger and clinically important population. Considering the high incidence of AF and IS risk in MI survivors, our findings emphasize the significance of MI as the only CHA_2_DS_2_-VASc point when considering whether to initiate anticoagulation in those with paroxysmal AF. AF develops in 20–40% of patients within the first week after CABG. Revascularization by CABG has been found to be associated with higher NOAF rates compared to patients who undergo PCI [16]. Furthermore, patients suffering from postoperative AF are at an increased risk of IS [17]. Despite the fact that most studies report AF episodes occurring during operation-related hospitalization, we found a similar and long-lasting pattern of NOAF occurrence after the index hospital visit. Our results show a 1.9-fold increase in cumulative NOAF incidence after CABG compared to PCI at one year and a 1.3-fold increase at ten years, whereas the highest cumulative NOAF incidence was recorded in patients assigned to noninterventional treatment (optimal medical therapy without PCI or CABG). It is reasonable to argue that patients undergoing CABG most likely suffer from multivessel disease more often than PCI patients and may have more comorbidities (beyond our hazard ratio adjustments), and thus healthier patients are selected for PCI. In addition, secondary prevention after MI is important when considering AF risk factor management (e.g., blood pressure, lipid levels, and diabetes) [18]. Consequently, there may also be differences in secondary preventive treatments prescribed by cardiologists vs. surgeons, which could have an influence on the observed differences in NOAF rates.

Patients with a history of MI are also at an increased risk of IS during the first three months after MI [19]. Previous data have identified several cardiogenic mechanisms behind this risk [20], most of which appear to be attenuated after the first month following MI, whereas the importance of AF increases thereafter [19]. According to our results, the hazard of IS is two times higher in patients who develop NOAF after MI compared to those who remain in a sinus rhythm. Previous studies have found IS to occur most commonly within the first year after AF diagnosis, while in up to 20% of IS survivors, the IS is the first manifestation of AF [21,22]. NOAF manifested as IS in 4% of our study patients. Of the patients who developed NOAF after MI, increasing age and prior cerebral ischemia were identified as long-lasting predictors of IS as they also remained significant at 10 years. Female sex was a risk factor within a year following MI, but not within ten years. This is probably because some individual IS risk factors are more significant IS predictors in women than in men, but the effect gets attenuated as competing IS risk factors in men, such as atherosclerosis, develop over time [23,24]. Therefore, early efforts to identify AF after MI are especially important in women. The need for careful patient follow-up is emphasized by the finding that IS and NOAF often occurred simultaneously, that is, without warning.

The incidence of IS was lowest in patients who underwent revascularization by PCI compared to those treated with CABG or medical therapy. This has also been suggested previously [25]. In addition, recent findings of Head et al. reported a significantly lower stroke rate five years after PCI vs. CABG in a pooled analysis of randomized studies comparing PCI and CABG, regardless of rhythm status [26]. Considering that NOAF occurs less frequently after PCI and patients usually have fewer comorbidities, the lower IS rate during long-term follow-up after PCI makes sense [27]. The antiplatelet regimens used after PCI [28] would not be expected to lower the risk of (cardio)embolic strokes, but they might lower the risk of atherosclerotic IS, which is more common in men and might also contribute to the observed sex difference within the first year following MI.

Our study has strengths and limitations. The major strength is the population-based design, which included nearly all patients with MI in Finland during a 14-year period. The major limitations are the retrospective design and use of registry data. We did not have access to more detailed clinical data and lacked blood pressure, laboratory, imaging, and angiographical findings. Although we used an extended version of a previously validated method to detect CHA_2_DS_2_-VASc components [9], it is possible that the true prevalence of some components, especially hypertension, could be underestimated in our data. Additionally, we did not have data on the evolvement of the CHA_2_DS_2_-VASc score during follow-up. Atrial fibrillation was detected using registries of specialist health care and death certificates, [11] but we did not have access to primary health care registries. A recent Finnish study showed that <4% of all AF patients during our study period would not be identified in the registries used in the current study [29]. The proportion of undetectable NOAF patients among MI survivors is likely to be even lower. In addition, information on OAC usage was not available in the current study. An inherent limitation of administrative registries is related to coding errors. However, the large number of patients makes it unlikely that these errors would significantly influence our main findings. We did not have data on the ethnic backgrounds of patients, but because the Finnish population is predominantly white, the generalizability of our results to more diverse populations may be limited.

## 5. Conclusions

Our study of 70,922 patients showed that the CHA2DS2-VASc-score is a feasible tool for long-term NOAF risk stratification in MI survivors. In addition, our results show that CABG is associated with increased long-term NOAF incidence after MI. The hazard of IS was roughly two-fold higher for patients with an NOAF diagnosis compared to those without. These results indicate that simple and clinically easily applicable risk stratification based on CHA_2_DS_2_-VASc scores may be helpful in future efforts aimed at NOAF detection to prevent IS.

## Figures and Tables

**Table 1:** Baseline features of study patients.

	Patients (*n* = 70,922) *n* (%)
Age (years) mean (SD)	68.2 (12.6)
<65	27,365 (38.6%)
65–74	19,155 (27.0%)
≥75	24,402 (34.4%)
Female sex	24,655 (34.7%)
Congestive heart failure	12,253 (17.3%)
Hypertension	35,252 (49.7%)
Diabetes	15,967 (22.5%)
Stroke/TIA/Thromboembolism	7191 (10.1%)
CHA_2_DS_2_-VASc score	
1	11,559 (16.3%)
2	13,755 (19.4%)
3	13,939 (19.6%)
4	12,601 (17.8%)
5	9640 (13.6%)
≥6	9438 (13.3%)
ST-elevation MI	27,589 (38.9%)
Revascularization	
None	26,423 (37.3%)
PCI	39,191 (55.3%)
CABG *	5308 (7.5%)

PCI = Percutaneous coronary intervention. CABG = coronary artery bypass grafting. TIA = Transient ischemic attack. * PCI was performed on 9.2% of patients treated with CABG.

**Table 2:** Cumulative incidence of new-onset atrial fibrillation after myocardial infarction at one- and ten-year follow-ups by baseline CHA_2_DS_2_-VASc score.

CHA_2_DS_2_-VASc Score	Cumulative Incidence *n* (%)		Unadjusted sHR (95%CI)		Adjusted sHR (95%CI)	
(*n*)	1 Year	10 Year	1 Year	10 Year	1 Year	10 Year
1 (11,559)	169 (1.5%)	673 (8.8%)	Reference	Reference	Reference	Reference
2 (13,755)	326 (2.4%)	1233 (14.0%)	1.63 (1.35–1.96)	1.61 (1.47–1.77)	1.60 (1.32–1.92)	1.59 (1.45–1.74)
3 (13,929)	491 (3.6%)	1837 (20.2%)	2.46 (2.06–2.93)	2.46 (2.25–2.69)	2.24 (1.96–2.78)	2.40 (2.20–2.62)
4 (12,601)	557 (4.6%)	2048 (24.1%)	3.12 (2.63–3.71)	3.14 (2.88–3.43)	2.91 (2.45–3.46)	3.04 (2.78–3.32)
5 (9640)	622 (6.7%)	1924 (28.7%)	4.64 (3.92–5.50)	4.05 (3.71–4.42)	4.24 (3.57–5.04)	3.89 (3.56–4.26)
≥6 (9438)	668 (7.5%)	1899 (27.9%)	5.21 (4.40–6.16)	4.22 (3.87–4.61)	4.66 (3.92–5.55)	4.03 (3.68–4.42)

Adjusted Fine–Gray models include revascularization, treatment in university hospital, ST elevation, and year of MI (only in one-year model). sHR = subdistribution hazard ratio.

**Table 3:** Proportion of new-onset atrial fibrillation (NOAF)-associated ischemic strokes (IS) during a 10-year follow-up of myocardial infarction patients without baseline AF.

CHA_2_DS_2_-VASc	Patients with IS	NOAF-Related IS
Score	*n*	*n* (%)
1	227	38 (16.7%)
2	447	76 (17.0%)
3	679	134 (19.7%)
4	751	194 (25.8%)
5	723	198 (27.4%)
≥6	964	213 (22.1%)

**Table 4:** Cumulative incidence of ischemic stroke in patients with new-onset atrial fibrillation after myocardial infarction at 1- and 10-year follow-ups by baseline CHA_2_DS_2_-VASc score.

CHA_2_DS_2_-VASc Score	Cumulative Incidence *n* (%)		Unadjusted sHR (95%CI)		Adjusted sHR (95%CI)	
(*n*)	1 Year	10 Year	1 Year	10 Year	1 Year	10 Year
1 (673)	25 (3.9%)	38 (7.1%)	Reference	Reference	Reference	Reference
2 (1233)	49 (4.3%)	76 (7.9%)	1.07 (0.66–1.72)	1.09 (0.74–1.61)	1.05 (0.65–1.70)	1.10 (0.74–1.61)
3 (1837)	88 (5.1%)	134 (9.1%)	1.26 (0.81–1.96)	1.26 (0.88–1.80)	1.24 (0.80–1.92)	1.25 (0.88–1.79)
4 (2048)	133 (6.9%)	194 (11.6%)	1.69 (1.11–2.59)	1.62 (1.15–2.29)	1.66 (1.08–2.54)	1.61 (1.14–2.27)
5 (1924)	137 (7.5%)	198 (12.5%)	1.84 (1.21–2.81)	1.76 (1.25–2.48)	1.78 (1.16–2.74)	1.72 (1.21–2.44)
≥6 (1899)	149 (8.2%)	213 (13.5%)	2.02 (1.33–3.08)	1.91 (1.36–2.69)	1.95 (1.27–2.99)	1.85 (1.31–2.62)

sHR = subdistribution hazard ratio. Adjusted Fine–Gray models include revascularization, treatment in university hospital, ST elevation, and year of MI (only in 1-year model).

## Data Availability

Not applicable.

## Supplementary Materials

The following supporting information can be downloaded at: https://www.mdpi.com/article/10.3390/jcm11237090/s1, Table S1. International classification of diseases version 10 (ICD-10) codes and prescription medication reimbursement codes of Social Insurance Institution of Finland (www.kela.fi) used for detection of CHA2DS2-VASc components; Table S2. Association of baseline features with cumulative incidence of new onset atrial fibrillation at 1-year and 10-years after myocardial infarction (MI); Table S3. Association of baseline features with cumulative incidence of ischemic stroke in patients with NOAF at 1-year and 10-year follow-ups after NOAF diagnosis; Figure S1. Study flowchart.
