# Supplementary material for: Association of CHA2DS2-VASc Score with Long-Term Incidence of New-Onset Atrial Fibrillation and Ischemic Stroke after Myocardial Infarction
Source: J Clin Med. 2022 Nov 30;11(23):7090. doi: 10.3390/jcm11237090 (PMC9739941; doi:10.3390/jcm11237090)
Supplement: Supplementary file 1 [file jcm-11-07090-s001.zip › jcm-2047169-supplementary.pdf]

Supplementary Materials

Data sources
The following nationwide, mandated-by-law registry data were collected and combined from all study patients:

- All hospital admissions, all outpatient visits in specialist medical care, all emergency room admissions (International Classification of Diagnosis / ICD-10), and operational codes (Nordic Classification of Surgical Procedures) collected from the CRHC registry. Received from the Findata. Available from Jan 1st 2004.
- Purchases of oral anticoagulants within 90 days prior to MI admission. Data including Anatomical Therapeutic Chemical (ATC) codes and purchase dates. Received from the Findata. Available from Jan 1st 2004.
- Entitlements to special reimbursements for prescription medications including entitlement codes and underlying ICD diagnoses. Received from the National Institute for Health and Welfare of Finland (Findata). Available from Jan 1st 1964.
- Mortality data including date and causes of death. Received from Statistics Finland. Available from Jan 1st 2005.

CHA_2DS_2-VASc components were recognized from the data of MI admission and the preceding data from all available registries. Revascularization procedures were recognized from MI admission.

Outcome definitions
- Atrial fibrillation (AF): ICD-10 code I48 as any diagnosis in the CRHC registry (including ward/hospital admissions, specialist medical care outpatient visits, and emergency room visits) or as any cause of death in death certificate.
- Ischemic stroke (IS): ICD-10 code I63 as primary discharge diagnosis in ward/hospital admission or as any cause of death on death certificate.
- New-onset AF-associated IS: New-onset AF diagnosed before IS or within 30 days after IS.

	ICD-10 codes	Prescription medication reimbursement codes
**Congestive heart failure**	I09.9, I11, I13, I25.5, I42, I43, I50, I51.7, P29.0	201, 283, 354, 381
**Hypertension**	I10-I13, I15	205
**Diabetes**	E10-E14	103, 215, 160, 162, 358, 171, 177, 285, 346, 371, 382
**Stroke / TIA / Thromboembolism**	G45.0, G45.1, G45.8, G45.9, G46.0-G46.6, I63-I66, I676, I693, I694, I26, I74	

Supplementary Table S1. International classification of diseases version 10 (ICD-10) codes and prescription medication reimbursement codes of Social Insurance Institution of Finland (www.kela.fi) used for detection of CHA_2DS_2-VASc components. Components were detected from the CRHC registry (available from January 1st 2004) and the registry of special mediation reimbursement permissions (including permission codes and underlying ICD diagnoses, available from Jan 1st 1964). ICD-10 codes were adapted with modifications from Webter-Clark M, Huang T-Y, Hou L, Toh S. Translating claims-based CHA_2DS_2-VaSc and HAS-BLED to ICD-10-CM: Impacts of mapping strategies. Pharmacoepidemiol Drug Saf. 2020;29:409–418.

Variable	1-year				10-year			
	Univariable		Multivariable		Univariable		Multivariable	
	**sHR**	**P-value**	**sHR**	**P-value**	**sHR**	**P-value**	**sHR**	**P-value**
Congestive heart failure	2.11 (1.95-2.29)	<0.0001	1.52 (1.39-1.66)	<0.0001	1.74 (1.66-1.82)	<0.0001	1.30 (1.24-1.37)	<0.0001
Hypertension	1.63 (1.51-1.76)	<0.0001	1.28 (1.18-1.39)	<0.0001	1.63 (1.56-1.69)	<0.0001	1.35 (1.29-1.41)	<0.0001
Age		<0.0001		<0.0001		<0.0001		<0.0001
<65	Reference		Reference		Reference		Reference	
65-74	2.00 (1.80-2.24)	<0.0001	1.80 (1.61-2.02)	<0.0001	2.13 (2.01-2.26)	<0.0001	1.97 (1.86-2.09)	<0.0001
≥75	3.32 (3.01-3.70)	<0.0001	2.68 (2.41-2.98)	<0.0001	2.98 (2.83-3.14)	<0.0001	2.59 (2.44-2.74)	<0.0001
Diabetes	1.33 (1.23-1.45)	<0.0001	1.06 (0.97-1.15)	0.217	1.30 (1.24-1.36)	<0.0001	1.05 (1.00-1.10)	0.041
Stroke / TIA / Thromboembolism	1.58 (1.43-1.76)	<0.0001	1.13 (1.02-1.26)	0.026	1.42 (1.34-1.51)	<0.0001	1.06 (0.99-1.13)	0.081
Female sex	1.37 (1.28-1.48)	<0.0001	0.99 (0.91-1.07)	0.757	1.31 (1.26-1.36)	<0.0001	0.97 (0.93-1.01)	0.185
STEMI	0.78 (0.72-0.85)	<0.0001	1.07 (0.98-1.16)	0.118	0.78 (0.75-0.81)	<0.0001	0.97 (0.93-1.02)	0.247
Revascularization		<0.0001		<0.0001		<0.0001		<0.0001
None	Reference		Reference		Reference		Reference	
PCI	0.55 (0.51-0.59)	<0.0001	0.75 (0.68-0.82)	<0.0001	0.70 (0.67-0.73)	<0.0001	0.94 (0.90-0.99)	0.014
CABG	1.10 (0.97-1.24)	0.138	1.40 (1.23-1.59)	<0.0001	1.01 (0.94-1.08)	0.806	1.24 (1.15-1.33)	<0.0001
Treatment in university hospital	0.90 (0.84-0.97)	0.006	1.02 (0.95-1.10)	0.537	0.86 (0.83-0.90)	<0.0001	0.95 (0.91-0.99)	0.012
Year of MI		0.140		0.124	-	-	-	-
2005-2009	Reference		Reference		-	-	-	-
2010-2013	1.05 (0.96-1.15)	0.327	1.10 (1.00-1.21)	0.042	-	-	-	-
2014-2018	0.95 (0.87-1.04)	0.285	1.05 (0.96-1.15)	0.273	-	-	-	-

Supplementary Table S2. Association of baseline features with cumulative incidence of new-onset atrial fibrillation at 1 year and 10 years after myocardial infarction (MI). PCI=Percutaneous coronary intervention. CABG=coronary artery bypass grafting. TIA=Transient ischemic attack. sHR=subdistribution hazard ratio.

Variable	1-year				10-year			
	Univariable		Multivariable		Univariable		Multivariable	
	**sHR**	**P-value**	**sHR**	**P-value**	**sHR**	**P-value**	**sHR**	**P-value**
Congestive heart failure	1.19 (0.94-1.34)	0.220	0.97 (0.85-1.24)	0.765	1.11 (0.96-1.28)	0.179	0.99 (0.84-1.15)	0.851
Hypertension	1.03 (0.87-1.21)	0.757	0.93 (0.78-1.10)	0.379	1.05 (0.92-1.21)	0.460	0.96 (0.83-1.11)	0.564
Age		<0.0001		0.0004		<0.0001		<0.0001
<65	Reference		Reference		Reference		Reference	
65-74	1.34 (1.03-1.75)	0.033	1.25 (0.96-1.65)	0.102	1.32 (1.06-1.64)	0.013	1.26 (1.01-1.57)	0.040
≥75	1.80 (1.42-2.29)	<0.0001	1.61 (1.25-2.07)	0.0002	1.69 (1.39-2.06)	<0.0001	1.55 (1.26-1.91)	<0.0001
Diabetes	1.13 (0.95-1.35)	0.182	1.14 (0.95-1.38)	0.161	1.10 (0.94-1.27)	0.229	1.09 (0.94-1.28)	0.267
Stroke / TIA / Thromboembolism	1.50 (1.22-1.85)	0.0001	1.42 (1.15-1.75)	0.001	1.50 (1.26-1.79)	<0.0001	1.40 (1.18-1.67)	0.0002
Female sex	1.38 (1.18-1.62)	<0.0001	1.22 (1.03-1.45)	0.019	1.26 (1.10-1.44)	0.001	1.13 (0.98-1.30)	0.091
STEMI	0.95 (0.80-1.13)	0.546	1.09 (0.92-1.30)	0.332	1.02 (0.88-1.17)	0.815	1.15 (0.99-1.33)	0.063
Revascularization		<0.0001		0.013		0.001		0.018
None	Reference		Reference		Reference		Reference	
PCI	0.69 (0.58-0.82)	<0.0001	0.79 (0.66-0.95)	0.014	0.75 (0.65-0.87)	<0.0001	0.81 (0.70-0.94)	0.007
CABG	0.95 (0.73-1.24)	0.709	1.12 (0.84-1.48)	0.442	0.89 (0.71-1.13)	0.343	1.23 (0.96-1.57)	0.990
Treatment in university hospital	0.89 (0.76-1.05)	0.159	0.95 (0.81-1.13)	0.577	0.99 (0.86-1.13)	0.829	1.04 (0.91-1.19)	0.555
Year of MI		0.171		0.132	-	-	-	-
2005-2009	Reference		Reference		-	-	-	-
2010-2013	0.91 (0.76-1.09)	0.301	0.90 (0.75-1.08)	0.264	-	-	-	-
2014-2018	0.81 (0.64-1.02)	0.073	0.79 (0.63-1.01)	0.055	-	-	-	-

Supplementary Table S3. Association of baseline features with cumulative incidence of ischemic stroke in patients with NOAF at 1-year and 10-year follow- ups after NOAF diagnosis. PCI=percutaneous coronary intervention. CABG=coronary artery bypass grafting. TIA=transient ischemic attack. sHR=subdistribution hazard ratio.

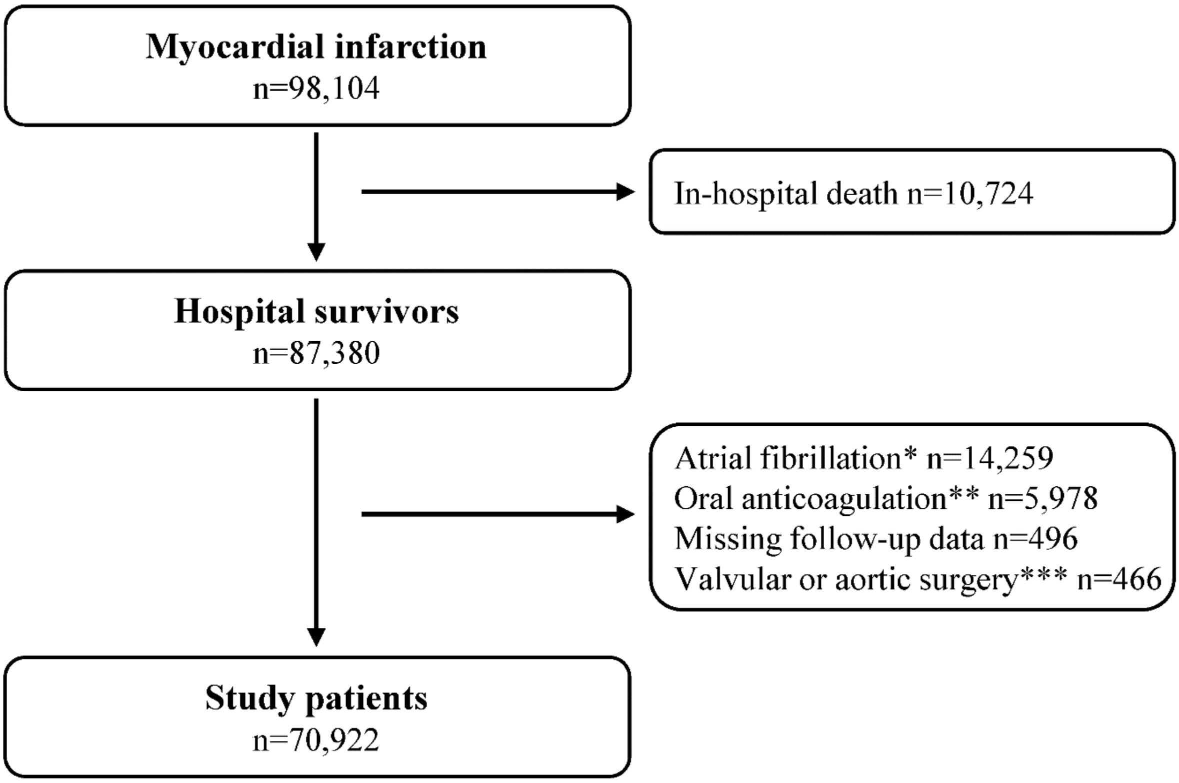

Supplementary Figure S1. Study flowchart. *before or during index MI admission, **before index MI, ***during index MI admission.
